# *Plasmodium falciparum* Malaria and Atovaquone-Proguanil Treatment Failure

**DOI:** 10.3201/eid1402.070945

**Published:** 2008-02

**Authors:** Rémy Durand, Virginie Prendki, Johann Cailhol, Véronique Hubert, Pascal Ralaimazava, Laurent Massias, Olivier Bouchaud, Jacques Le Bras

**Affiliations:** * Hôpital Avicenne, Bobigny, France; † Université Léonard de Vinci, Bobigny, France; ‡ Hôpital Bichat Claude Bernard, Paris, France; § Université René Descartes, Paris, France

**Keywords:** *Plasmodium falciparum*, atovaquone-proguanil, therapeutic failure, dispatch

## Abstract

We noticed overrepresentation of atovaquone-proguanil therapeutic failures among *Plasmodium falciparum*–infected travelers weighing >100 kg. We report here 1 of these cases, which was not due to resistant parasites or impaired drug bioavailability. The follow-up of such patients should be strengthened.

Fewer than 25 cases of falciparum malaria that failed to respond to atovaquone-proguanil (A-P) have been noted in published articles since the 1996 registration of Malarone (GlaxoSmithKline, Marly-le-Roi, France) (*1**,**2*). Well-documented cases that were not attributed to suboptimal dosage or impaired bioavailability essentially due to vomiting, diarrhea, or both, showed atovaquone-resistant parasites in the recrudescent isolate, with Y268S or Y268N cytochrome b mutations (*2**–**10*). We report the case of treatment failure that was not due to resistant parasites or impaired drug bioavailability.

## The Case

During February 2007, a 39-year-old man who was born in Africa but lived in France since 1996 traveled to Kinshasa, Democratic Republic of Congo, for a 1-month vacation in which he visited friends and relatives. He was 6 feet tall with strong musculature and weighed 115 kg (body mass index = 34.3). He did not use chemoprophylaxis and did not take antimalarial self-treatment before seeking medical advice. Two days after returning to France, a fever and other signs of uncomplicated malaria attack developed (chills, arthralgia, asthenia) without vomiting. Three days after onset of symptoms, the patient sought care at the Avicenne Hospital, Bobigny, France. Blood smears performed at admission showed 1.6% *Plasmodium falciparum* parasitemia. The patient was hospitalized and immediately treated with the standard dosage of A-P (Malarone, 4 tablets each day for 3 days given with the main meal; each tablet contains 250 mg of atovaquone and 100 mg of proguanil hydrochloride). The patient experienced no vomiting or diarrhea. His fever abated by day 3 of treatment, and malaria smears at that time showed 0.07% of morphologically altered parasites (these figures are not uncommon because A-P is known to act relatively slowly). The patient was discharged on day 3. The patient did not return until his scheduled appointments for control of parasitemia on days 7 and 28. The patient was apyretic, and parasitemia was negative by day 7 on thin and thick blood smears. On day 28, the patient was apyretic, but thin and thick smears showed *P. falciparum* trophozoites (0.001% parasitemia on thin smear and 16 trophozoites per 1,000 leukocytes on thick smear). The patient was then successfully retreated with 650 mg quinine base orally 3× daily for 7 days.

Day 0 in vitro phenotype showed parasite susceptibility to atovaquone, with a 50% inhibitory concentration (IC_50_) value of 10 nmol/L (in vitro resistance threshold >40 nmol/L [*11*]). Day 28 in vitro susceptibility was not assayed because of insufficient parasite density. DNA sequencing showed that both day 0 and day 28 isolates had wild-type sequence of cytochrome b. Genotyping of the *pfdhfr* gene showed that the 3 major *pfdhfr* mutations (at position 51, 59, and 108) associated with cycloguanil resistance were found in isolates from day 0 and day 28. The number and the proportions of genotypes within isolates were determined by a fragment analysis method based on the polymorphism of the gene encoding merozoite surface protein-2 (*12*). Day 0 and day 28 isolates contained the same majority genotype, with the 727-bp msp-2 allele representing >80% of isolates. This parasite population analysis did not show the selection of a minority-resistant genotype by A-P treatment. These results did not show either the emergence of mutant codon 268 cytochrome b within the 727-bp msp-2 dominant genotype. High-performance liquid chromatography on day 3 of treatment (performed 20 h after the last drug intake) showed an atovaquone plasma concentration of 3.1 μg/mL. High interpatient variability has been reported, but this value showed initial adequate drug concentration and excluded impaired bioavailability (*13*).

Thus, this patient, who had correctly taken A-P tablets with food and did not vomit, showed correct plasma drug concentration on day 3 but did not show clearance of A-P–susceptible parasites on day 28, although he was asymptomatic. Reinfection was excluded because the patient was treated after returning to France (which is a non–malaria-endemic area). All these data suggest that the standard drug regimen led to suboptimal dosage in this patient. Apparently correct initial atovaquone concentration on day 3 did not predict the outcome of treatment because A-P acts slowly, and most reported A-P therapeutic failures were late failures. Drug interactions that could have lowered A-P plasmatic concentration were excluded because, other than A-P, the patient received only acetaminophen.

## Conclusions

The most plausible cause of this late therapeutic failure is the relatively insufficient dosage due to increased oral clearance and volume of distribution of atovaquone in this patient who weighed >100 kg. According to the relationships between oral clearance of atovaquone and weight, and between volume of distribution and weight, these parameters increase by 40% in comparison to those in a patient of 70 kg (*13*). The effect may be less marked for proguanil because, unlike atovaquone, it becomes concentrated in the erythrocytes. However, proguanil likely does not act by itself in A-P association but only facilitates the atovaquone activity (*14*). Likewise, the observed *pfdhfr* triple mutant likely had no effect on the intrinsic activity of proguanil but only reflected the high frequency of this haplotype in West Africa.

Another hypothesis is that the treatment failure was due to resistant parasites not related to the cytochrome b mutations. Some rare therapeutic failures remain unexplained by phenotypic or genotypic data. Such a parasite should have emerged during treatment, because the IC_50_ of the day 0 isolate was susceptible. We know of only 1 other reported case of A-P treatment failure not associated with cytochrome b mutations and not related to incorrect dosage or impaired bioavailability (*15*). In the case reported by Wichmann et al. (*15*), clonal analysis of pre- and posttreatment isolates was not performed, which limited the interpretation of DNA sequencing; the weight of the patient was not mentioned.

Most previously reported A-P therapeutic failures were late failures: patients sought care or parasitemia was detected >3 weeks after first day of treatment in 13 of 21 cases (*2**–**5**,**7**–**9**,**15*). These data underline the usefulness of the day 28 appointment in detecting late A-P therapeutic failures. In addition, this appointment may provide the opportunity to detect asymptomatic parasitemia (*2*), as occurred in the present case.

We noticed an overrepresentation of A-P therapeutic failures among patients who weighed >100 kg. In a series of 347 *P. falciparum*–infected travelers, 3 of 12 patients who weighed 100–115 kg exhibited therapeutic failure while receiving the standard A-P regimen (only 2 therapeutic failures were reported among the other 335 patients who weighed <100 kg). Two of these patients >100 kg showed mutant parasites on the date treatment failure was recognized (*2*); the third treatment failure (the present one) was due to susceptible parasites. Thus, relative suboptimal dosage of the standard A-P regimen in patients >100 kg led to either failure to control susceptible parasites or emergence of resistant ones. DNA point mutations conferring atovaquone resistance may emerge more easily in patient who have received suboptimal dosage. However, not all patients weighing >100 kg seen in our center had A-P therapeutic failures. Eleven of these 12 patients were from Africa and could have possessed residual immunity, which could have helped them clear parasites.

A-P is safe and therapeutic failure remains rare. Nevertheless, the overrepresentation of failures in patients >100 kg argues for strengthening the follow-up monitoring of those patients. Moreover, the weight of a patient could be taken into account in the dosage of A-P prescribed for similar cases in the future.
